# Cell‐Based Phenotypic and Target Engagement Assays for the Identification of Small Molecules that Enhance Microglial Phagocytosis

**DOI:** 10.1002/alz70859_106545

**Published:** 2025-12-26

**Authors:** Emily R Mason, Omar El Jordi, Shaoyou Chu, TREAT‐AD Center, IUSM

**Affiliations:** ^1^ Indiana University School of Medicine, Indianapolis, IN USA

## Abstract

**Background:**

Enhancement of microglial phagocytosis activity is being pursued as a potential therapeutic strategy for Alzheimer’s Disease (AD). Assays evaluating small molecule compound effects on microglial phagocytosis and target binding specificity in live cells may help drug discovery along this strategy, such as our projects seeking SHIP1 inhibitors and PLCG2 activators.

**Methods:**

We developed paired cell‐based assay platforms to evaluate small molecule effects on microglial phagocytosis activity and cell health as well as target engagement for specificity. The phenotypic high‐content imaging assay is used to evaluate microglial phagocytosis activity and cell health. Microglia (HMC3, BV2 or primary mouse microglia) are plated in 384‐well plates, treated with compounds on day 2, seeded with pHrodo labeled myelin/cell membrane debris on day 3, and cells stained with Hoechst 33342 DNA dye and imaged on day 4. Phagocytosis and cell health are evaluated by quantifying phagocytosis vesicle fluorescence, cell counting and average nuclear intensity. Target binding and specificity of compounds are assessed using a series of cell‐based thermal shift assays (CETSAs) that utilize the HiBiT/LgBiT reconstituted NanoLuc technology. Intact human (HEK293T or HMC3) cells stably overexpressing the full‐length target protein tagged with HiBiT (an 11‐amino acid tag) are plated in 96‐well PCR plates, treated with compounds for one hour, followed by brief heating at target specific Tm, cell lysis, addition of LgBiT and NanoLuc substrate, and luminescence reading. Alterations in the luminescence signal indicate compound binding to the HiBiT‐tagged target protein.

**Results:**

Implementation of these assays into our drug discovery team’s workflow has streamlined compound selection, improving efficiency in identifying and optimizing lead compounds that enhance microglial phagocytosis via selective interaction with and modulation of targeted proteins, e.g. inhibition of SHIP‐1 or activation of PLCG2, in live cells. Active compounds for each project were discovered with these assays.

**Conclusions:**

This combination of phenotypic and target engagement cell‐based assays is widely applicable to drug discovery projects. Our studies demonstrated the feasibility of combining phenotypic assays with target engagement assays to ensure compound specificity for drug discovery projects. This strategy can be useful when specific signaling measurement is unavailable or difficult to implement for some targets.

